# TRPC3 determines osmosensitive [Ca^2+^]_i_ signaling in the collecting duct and contributes to urinary concentration

**DOI:** 10.1371/journal.pone.0226381

**Published:** 2019-12-18

**Authors:** Viktor N. Tomilin, Mykola Mamenko, Oleg Zaika, Guohui Ren, Sean P. Marrelli, Lutz Birnbaumer, Oleh Pochynyuk

**Affiliations:** 1 Department of Integrative Biology and Pharmacology, the University of Texas Health Science Center at Houston, Houston, Texas, United States of America; 2 Department of Physiology, Medical College of Georgia, Augusta University, Augusta, Georgia, United States of America; 3 Department of Neurology, the University of Texas Health Science Center at Houston, Houston, Texas, United States of America; 4 Neurobiology Laboratory, National Institute of Environmental Health Sciences, Research Triangle Park, North Carolina, United States of America; 5 Institute of Biomedical Research (BIOMED), School of Medical Sciences, Catholic University of Argentina, Buenos Aires, Argentina; Indiana University School of Medicine, UNITED STATES

## Abstract

It is well-established that the kidney collecting duct (CD) plays a central role in regulation of systemic water homeostasis. Aquaporin 2 (AQP2)-dependent water reabsorption in the CD critically depends on the arginine vasopressin (AVP) antidiuretic input and the presence of a favorable osmotic gradient at the apical plasma membrane with tubular lumen being hypotonic compared to the cytosol. This osmotic difference creates a mechanical force leading to an increase in [Ca^2+^]_i_ in CD cells. The significance of the osmosensitive [Ca^2+^]_i_ signaling for renal water transport and urinary concentration remain unknown. To examine molecular mechanism and physiological relevance of osmosensitivity in the CD, we implemented simultaneous direct measurements of [Ca^2+^]_i_ dynamics and the rate of cell swelling as a readout of the AQP2-dependent water reabsorption in freshly isolated split-opened CDs of wild type and genetically manipulated animals and combined this with immunofluorescent detection of AVP-induced AQP2 trafficking and assessment of systemic water balance. We identified the critical role of the Ca^2+^-permeable TRPC3 channel in osmosensitivity and water permeability in the CD. We further demonstrated that TRPC3 -/- mice exhibit impaired urinary concentration, larger urinary volume and a greater weight loss in response to water deprivation despite increased AVP levels and AQP2 abundance. TRPC3 deletion interfered with AQP2 translocation to the plasma membrane in response to water deprivation. In summary, we provide compelling multicomponent evidence in support of a critical contribution of TRPC3 in the CD for osmosensitivity and renal water handling.

## Introduction

Kidneys play a central role in systemic water balance by excreting urine with a highly variable osmolarity depending on hydration status [1, 2]. Antidiuretic hormone, arginine vasopressin (AVP) augments water permeability of the collecting duct (CD) principal cells by driving translocation and incorporation of the aquaporin 2 (AQP2) containing vesicles to the apical plasma membrane and, at a longer timescale, by increasing AQP2 expression [3, 4]. Inability of the CD cells to respond to AVP signal due to genetic or acquired defects causes Nephrogenic Diabetes Insipidus (NDI) leading to excretion of a copious volume of urine, dehydration and polydipsia [5, 6]. It is generally recognized that activation of the G-protein coupled vasopressin receptors type 2 (V2R) stimulates production of cyclic adenosine monophosphate (cAMP) to increase AQP2 trafficking and synthesis through an intricate and multifactorial signaling network, including cAMP-activated protein kinase A (PKA), cAMP responsive binding protein (CREB), and calcineurin–nuclear factor of activated T cells (NFAT) to name a few [7–10].

AVP stimulation primes the CD to reabsorb water, but this occurs only when positive osmotic difference exists between the cytosol and tubular fluid. This osmotic gradient exerts a mechanical stress of the apical membrane thereby leading to increased cell volume (swelling) and elevated [Ca^2+^]_i_ [11]. It is appreciated that mechanical forces arising from variations in tubular flow and osmolarity serve as important determinants of numerous physiologically relevant processes in epithelial cells, including transport of water and electrolytes, proliferation, polarization, etc. [12, 13]. Transient receptor potential (TRP) channels can serve as mediators of a variety of environmental stimuli, such as temperature, various chemical and mechanical inputs [14]. TRP channel activation drives Ca^2+^ entry from the extracellular medium leading to the elevation of [Ca^2+^]_i_ to initiate cellular responses [14, 15]. Expression and functional activity of several TRP channels, including TRPC3, TRPC6, and TRPV4, have been reported in the native CD cells and CD-derived cultures [16, 17]. Accumulated evidence has demonstrated that TRPV4 is indispensable for flow-induced [Ca^2+^]_i_ elevations [17–19]. However, TRPV4 -/- mice do not demonstrate measurable defects in the renal water handling [20, 21] indicating that distinct molecular mechanisms are involved in sensing changes in flow and osmolarity in the CD.

It has been reported that, in addition to cAMP, increased [Ca^2+^]_i_ plays an important role in synthesis and trafficking of AQP2 in the CD cells [22–25]. Interestingly, systemic AVP infusion causes translocation of TRPC3, but not TRPC6, channels to the apical plasma membrane by stimulating the V2R-adenylyl cyclase (AC)/cAMP/ PKA signaling cascade [26, 27]. Recently determined cryo-EM structure of the full-length human TRPC3 revealed an unusually long transmembrane domain 3 (S3) region forming an extracellular pocket-like domain, which could potentially serve as a sensor of mechanical stimuli [28]. However, it is unknown whether TRPC3 mediates osmosensitive [Ca^2+^]_i_ signaling and whether it contributes to the regulation of the osmotically-driven water reabsorption in the CD cells.

In this study, we employed freshly isolated CDs to demonstrate a functional coupling between osmosensitive [Ca^2+^]_i_ signaling and water permeability and established an important role of the TRPC3 channel in both processes. We further uncovered that TRPC3 deficiency distorts subcellular AQP2 localization at the baseline and during antidiuresis and causes an impaired urinary concentrating ability and notable disturbances of systemic water balance, which cannot be compensated by high AVP levels. We further speculate that TRPC3-based pharmacological intervention might be of use to regulate urine production and osmolarity.

## Materials and methods

### Reagents and animals

All chemicals and materials were from Sigma (St. Louis, MO, USA), VWR (Radnor, PA, USA), Fisher (Waltham, MA, USA), and Tocris (Ellisville, MO, USA) unless noted otherwise and were at least of reagent grade. Animal use and welfare adhered to the NIH Guide for the Care and Use of Laboratory Animals following protocols reviewed and approved by the Animal Care and Use Committees of the University of Texas Health Science Center at Houston. For experiments, 6–10 weeks old C57BL/6 (WT, purchased from Charles Rivers Laboratories, Wilmington, MA), TRPC3 -/- (having C57BL/6 background) and TRPV4 -/- (having C57BL/6 background) mice were used. The usage of TRPC3 -/- and TRPV4 -/- mice have been described previously [20, 29]. Animals were maintained on standard rodent regimen (7012 Teklad LM-485 Diet; Envigo, Cambridgeshire, UK).

### Systemic measurements

Mice were acclimated for few days in metabolic cages (3600M021; Techniplast, West Chester, PA, USA). Following acclimation and baseline measurements of urinary parameters, mice were water deprived for 24 hours, as we did previously [25]. Tails were clipped with sterilized scissors to collect 50 μl blood into heparinized micro-hematocrit capillary tubes (Fischer, Cat. # 22-362-566) before and after water deprivation. Urine and plasma osmolarity was measured in duplicates using freezing point depression osmometer Model 3320 (Advanced Instruments, Norwood, MA, USA). Urinary creatinine concentration was assessed with QuantiChrom Creatinine Assay Kit (DICT-500; BioAssay Systems, Hayward, CA, USA) utilizing the improved Jaffe method, as we did before [30]. Urinary AVP levels were assayed with Vasopressin Arg^8^-Vasopressin ELISA kit (Enzo Life Sciences, Farmingdale, NY, USA) following the manufacturer’s protocols. Urinary Ca^2+^ concentration was measured using a Jenway PFP7 Flame photometer (Bibby Scientific, Burlington, NJ, USA).

### Isolation of individual collecting ducts (CDs)

The procedure for isolation of the CDs from WT and mutant mice suitable for fluorescent-based [Ca^2+^]_i_ and cell volume measurements closely followed the protocols previously published by our group for patch clamping and intracellular Ca^2+^ imaging [31, 32]. Kidneys were cut into thin slices (< 1 mm), further placed into an ice-cold bath solution contained (in mM): 155 Mannitol, 60 NaCl, 5 KCl, 1 CaCl_2_, 2 MgCl_2_, 5 glucose and 10 HEPES (300 mOsm, pH 7.35). CDs were visually identified by their morphological features (pale color; coarse surface and, in some cases, bifurcations) and were mechanically isolated from kidney slices by micro-dissection using watchmaker forceps under a stereomicroscope. Isolated CDs were attached to 5 x 5 mm cover glasses coated with poly-L-lysine. A cover-glass containing a CD was placed in an experimental chamber mounted on an inverted Nikon Eclipse Ti microscope and perfused with the aforementioned bath solution at room temperature. CDs were split-opened with two sharpened micropipettes, controlled with different micromanipulators, to gain access to the apical membrane. The CDs were used within 2 hours of isolation.

### [Ca^2+^]_i_ and cell volume measurements

Intracellular calcium levels were continuously monitored in individual cells within a split-opened area of freshly isolated CDs using Fura 2 fluorescence imaging as described previously [32–34]. Briefly, split-opened CDs were loaded with Fura-2 by incubation with 2 μM Fura-2/AM in a bath solution for 40 min at room temperature. Subsequently, tissue samples were washed and incubated for additional 10 minutes prior to experimentation. CDs were placed in an open-top imaging study chamber (RC-26GLP; Warner Instruments, Hamden, CT, USA) constantly perfused at 1.5 ml/min with a bottom coverslip viewing window and the chamber attached to the microscope stage of a Nikon Ti-S Wide-Field Fluorescence Imaging System (Nikon Instruments, Melville, NY, USA) integrated with Lambda XL light source (Sutter Instrument, Novato, CA, USA) and QIClick 1.4 megapixel monochrome CCD camera (QImaging, Surrey, BC, Canada) via NIS Elements 4.3 Imaging Software (Nikon Instruments, Melville, NY, USA). Cells were imaged with a 40X Nikon Super Fluor objective and regions of interest (ROIs) were drawn for individual cells. For [Ca^2+^]_i_ measurements, the Fura 2 fluorescence intensity ratio was determined by excitation at 340 nm and 380 nm and calculating the ratio of the emission intensities at 511 nm in the usual manner every 5 seconds. The changes in the ratio are were converted into changes in intracellular calcium, as documented previously [35]. For monitoring of cell volume, changes in Fura 2 fluorescent intensity with 360 nm excitation, the Ca^2+^-independent isosbestic point [36], were detected. The signals were normalized to the initial values for each cell individually. Minor decrease in Fura 2 signal due to bleaching (less than 10% for 10 min) was corrected by a linear fit, as described previously [37]. In average, 4–6 individual CDs from 3 different mice were used for each experimental set. Experiments were conducted in a constantly perfused chamber at 1.5 ml/min using bath solution: (in mM): 155 Mannitol 60 NaCl, 5 KCl, 1 CaCl_2_, 2 MgCl_2_, 5 glucose and 10 HEPES (300 mOsm; pH 7.35). Hypertonic (400 mOsm) and hypotonic (220 mOsm) media were prepared by increasing and decreasing mannitol concentration to 255 mM and 80 mM, respectively. For experiments testing contribution of extracellular Ca^2+^, CaCl_2_ was omitted and 5 mM of Ca^2+^ chelator, EGTA (ethylene glycol-bis(β-aminoethyl ether)-N,N,N′,N′-tetraacetic acid) was added. Osmolarities were verified using a freezing point depression osmometer (Model 3320; Advanced Instruments, Norwood, MA, USA) before the actual experiments. The rate of cell swelling was calculated as a linear slope of the initial changes in Fura 2 fluorescence intensity at 360 nm excitation upon application of osmotic stimuli for each cell individually. For hypotonicity-induced [Ca^2+^]_i_ responses, the peak was calculated as the difference of the highest intracellular calcium concentration and the respective baseline.

### Western blotting

Immediately after dissection kidneys were placed on ice, decapsulated and homogenized in 5 volumes of hypotonic lysis buffer: 50 mM TrisCl, 5 mM EDTA, 1% Triton X-100 (pH = 7.5) supplemented with Complete Mini protease and PhosSTOP phosphatase inhibitor cocktails (Roche Diagnostics, Indianapolis, IN, USA). The homogenates were centrifuged at 1000 g for 15 min at +4 ºC and the sediment was discarded. Protein concentration was determined with a Bradford assay using bovine serum albumin as a standard. The samples (20 μg/lane) were separated on 10% polyacrylamide gels at 150 V for 100 min and transferred to a nitrocellulose membrane for 110 min at 100 V. Subsequently the nitrocellulose membrane was incubated with primary antibodies for 2 hours at room temperature. The primary antibodies were anti-AQP2 (1:1500, Alomone Labs, Israel; Cat. # AQP2-002), anti-AQP4 (1:1000, Alomone Labs, Israel; Cat. # AQP-004) and anti-β-actin (1:5000, Abcam, UK; Cat. # ab8227). Upon washout (3 times for 10 min in TBS-Tween) the membrane was incubated with peroxidase-conjugated goat anti-rabbit (1:10000, Jackson ImmunoResearch Laboratories, USA) secondary antibodies for 1 hour 30 min at room temperature. For AQP4, membranes were stained with Ponceau red (0.1% in 5% acetic acid) for 60 min. Blots were quantified using ImageJ 1.50 software (NIH, USA). The intensities of the studied protein bands were normalized to the intensities of the corresponding β-actin bands used as a loading control or overall Ponceau signal in a line.

### Immunofluorescent confocal microscopy in kidney sections

Kidneys were fixed in 10% neutral buffer formalin for 24 hours at +4°C and soaked in 0.9 M sucrose buffer for 4 hours for cryopreservation. Kidneys were further placed in O.C.T. Compound (Sakura Tissue-Tek 4583, Torrance, CA, USA), snap frozen on dry ice and placed into -80°C. Transverse cut 6 μm thick sections were made on CM 1850 cryostat (Leica, Buffalo Grove, IL, USA). The sections were allowed to warm to room temperature, washed 3 times in phosphate buffer saline (PBS), permeabilized with 1% SDS for 10 minutes following washout with PBS and blockade of non-specific binding with 1% BSA (Jackson Immunoresearch, West Grove, PA, USA) for an hour at room temperature. Sections were further incubated overnight with anti-AQP2 (1:4000, Alomone Labs, Israel; Cat. # AQP2-002) followed by secondary antibodies Alexa 488 (1/1000 Invitrogen; Cat.# A11070) or Alexa 594 (1/1000 Jackson Immunoresearch, West Grove, PA, USA Cat.# 111-587-003). Nuclei were stained with DAPI (0,5 μg/ml) for 10 minutes at room temperature. The labeled kidney sections were mounted with Fluoromount-G mounting media (SouthernBiotech, Birmingham, AL, USA) and imaged with a Nikon A1R confocal microscope, as we did similarly before [17, 25]. In brief, samples were excited with 405 and 488 or 561 nm laser diodes and emission captured with a 16-bit Cool SNAP HQ^2^ camera (Photometrics) interfaced to a PC running NIS elements software.

### Data analysis

All summarized data are reported as mean ± SEM. Statistical comparisons were made using one-way ANOVA with post-hoc Tukey test or one-way repeated measures ANOVA with post hoc Bonferroni test (for paired experiments within the same group). P value less than 0.05 was considered significant.

## Results

### Distinct molecular pathways mediate [Ca^2+^]_i_ responses to elevated flow and hypotonicity in CD cells

**Fig 1:**
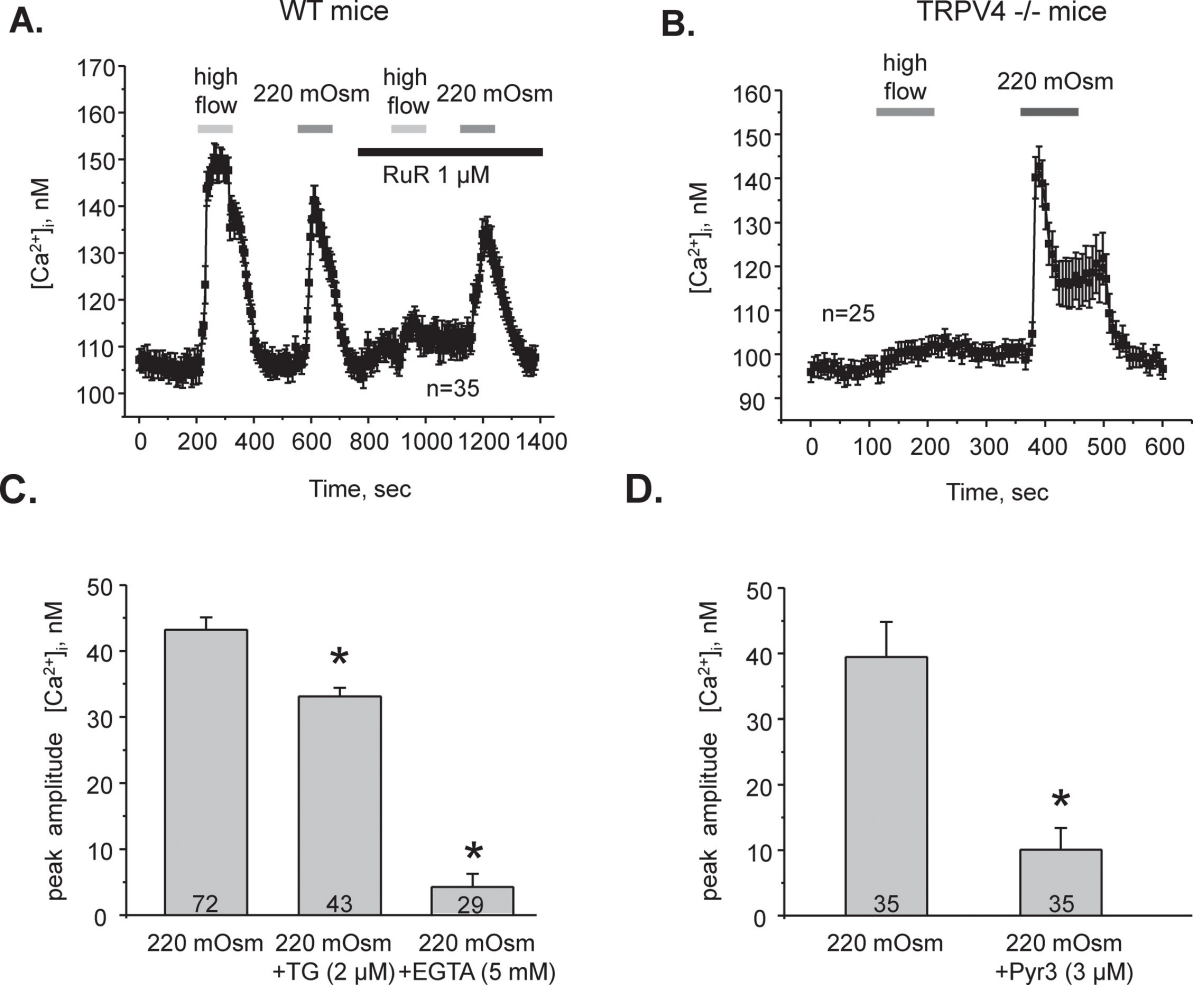
Hypotonic-mediated [Ca^2+^]_i_ signals require Ca^2+^ influx via TRPC3 in CD cells. (**A)** The average time course of [Ca^2+^]_i_ changes in response to 10X elevations in perfusion rate from 1.5 ml/min to 15 ml/min (high flow) and application of hypotonic medium (220 mOsm) in the absence and in the presence of a TRPV4 inhibitor, ruthenium red (RuR, 1 μM) in individual CD cells within split-opened area. The treatment times are indicated by the respective bars on the top. The number of individual experiments is shown (N = 3 mice were tested). (**B)** The average time course of [Ca^2+^]_i_ changes in response to high flow and hypotonicity in the CD cells from TRPV4 -/- mice (n–number of individual tested CD cells, N = 3 mice). **(C)** Summary graph of the peak amplitude of [Ca^2+^]_i_ responses induced by hypotonic 220 mOsm medium in the control, after pretreatment with the endoplasmic reticulum Ca^2+^ pump SERCA inhibitor thapsigargin (2 μM, 10 min), and after removing extracellular Ca^2+^ (5 mM EGTA). **(D)** Summary graph of the peak amplitude of [Ca^2+^]_i_ responses induced by hypotonic 220 mOsm medium in the control and after pretreatment with a TRPC3 channel blocker, Pyr3 (3 μM for 5 min). *—significant decrease versus 220 mOsm. N = 3 mice were used for the comparison.

It is known that reduced extracellular osmolarity exerts a mechanical stretch of the plasma membrane and causes a rise of [Ca^2+^]_i_. We and others demonstrated that the mechanosensitive Ca^2+^-permeable TRPV4 channel is essential for elevations in [Ca^2+^]_i_ in response to high flow over the apical plasma membrane in the CD cells [17, 18, 38, 39]. At the same time, it is not known whether high flow-induced shear stress and hypotonicity raise [Ca^2+^]_i_ by the same pathway. Thus, we first monitored changes in [Ca^2+^]_i_ in individual cells in freshly isolated split-opened CDs in response to a shear stress of approximately 3 dyn/cm^2^ (an abrupt increase in perfusion rate of the recording chamber from 1.5 ml/min to 15 ml/min, as we did previously [17]) and application of hypotonic medium (a decrease in osmolarity from 300 mOsm to 220 mOsm (see Methods) by decreasing mannitol concentration, while keeping concentration of electrolytes to avoid changes in electro-chemical gradients). Both stimuli elicited comparable [Ca^2+^]_i_ responses (Fig 1A). However, inhibition of TRPV4 activity with Ruthenium Red (RuR: 1 μM for 3 min) abolished cellular responses to increased flow, as expected, but not to decreased osmolarity (Fig 1A). To account for potential non-specific actions of RuR, we next monitored responses to high flow and hypotonicity in split-opened CDs from TRPV4 -/- mice. Flow-induced [Ca^2+^]_i_ responses were abolished in TRPV4 -/- mice (Fig 1B). In contrast, we observed a prominent [Ca^2+^]_i_ elevation in response to hypotonicity (Fig 1B). Altogether, these results strongly suggest that different molecular pathways are used to increase [Ca^2+^]_i_ to shear stress and hypotonic swelling in CD cells.

### CD responses to decreased osmolarity involve Ca^2+^ influx across the plasma membrane via TRPC3

Both intracellular and extracellular Ca^2+^ may serve as a source of [Ca^2+^]_i_ response to hypotonicity in CD cells. Blockade of the endoplasmic reticulum Ca^2+^ pump SERCA with thapsigargin (2 μM for 10 min) exerted only a mild (~20%), whilst significant, inhibitory effect on [Ca^2+^]_i_ responses to application of hypotonic (220 mOsm) medium (Fig 1C). In contrast, removal of extracellular Ca^2+^ (5 mM EGTA) precluded elevations in [Ca^2+^]_i_ induced by hypotonicity (Fig 1C). We concluded that direct Ca^2+^ entry through Ca^2+^-conducting pathways, such as ion channels, plays a dominant role in [Ca^2+^]_i_ elevations during hypotonic stimuli in CD cells.

In addition to TRPV4, other Ca^2+^-permeable channels, including TRPC3 and TRPC6, are known to be expressed in the CD cells [16, 40]. Interestingly, TRPC3 but not TRPC6 translocates to the apical plasma membrane in CD cells in response to AVP, the hormone governing osmotically-driven AQP2-dependent water reabsorption in the CD [26]. Pretreatment with a TRPC3 inhibitor, Pyr3 (3 μM, for 5 min) drastically diminished the amplitude of [Ca^2+^]_i_ elevations in response to application of 220 mOsm medium (Fig 1D), indicating that osmosensitive [Ca^2+^]_i_ responses occur predominantly in a TRPC3-dependent manner in CD cells.

### Independent monitoring of [Ca^2+^]_i_ dynamics and cell volume with Fura-2 in CD cells

**Fig 2:**
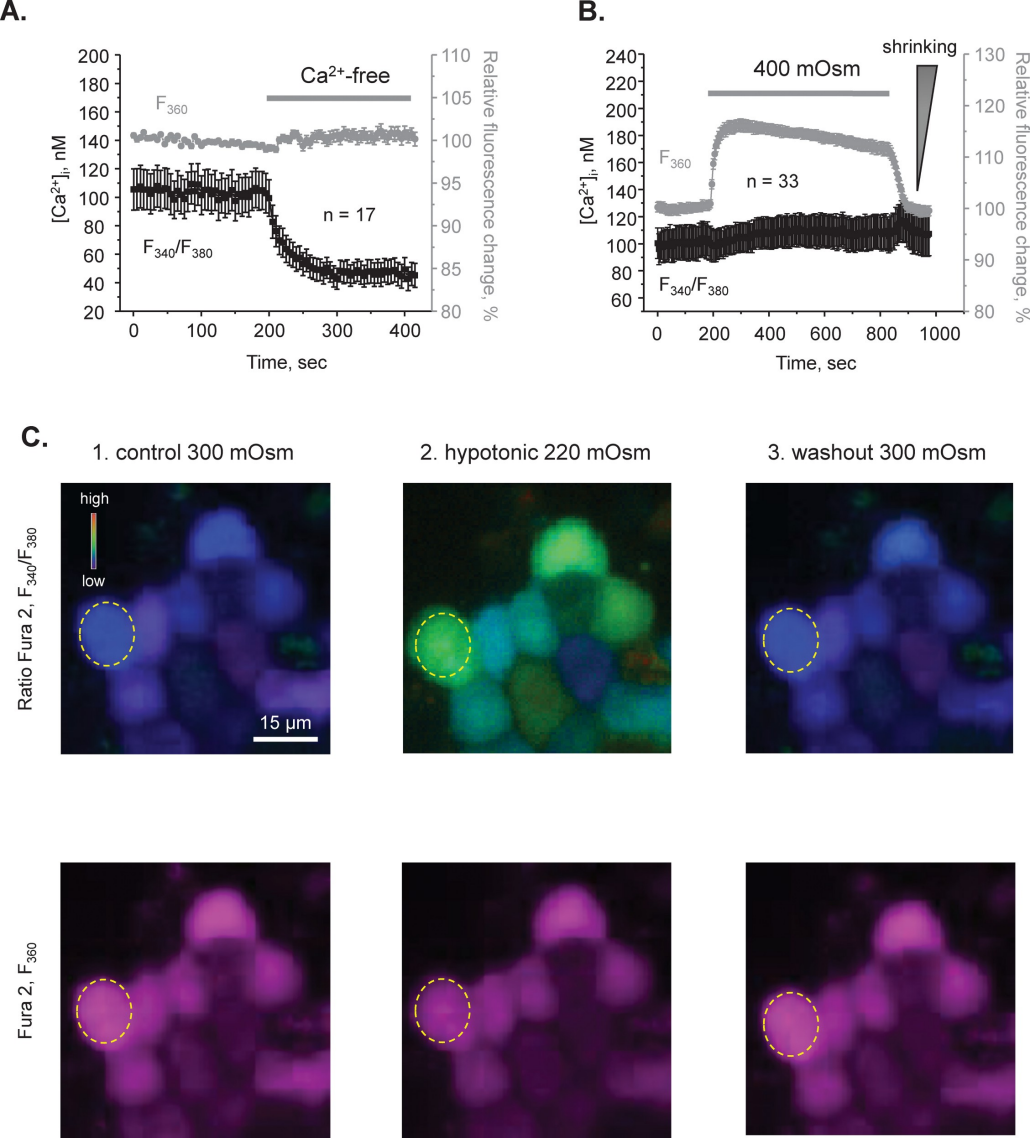
Independent measurements of [Ca^2+^]_i_ dynamics and cell volume changes in freshly isolated split-opened CDs. **(A)** The average time courses of changes in [Ca^2+^]_i_ (black) and normalized Fura 2 fluorescent with excitation at 360 nm, Ca^2+^-independent isosbestic point, as a readout of relative cell volume changes (grey) in split-opened CDs in the control and upon application of Ca^2+^-free medium (shown with a bar on the top). **(B)** The average time courses of changes in [Ca^2+^]_i_ (black) and relative cell volume changes in response to application of hypertonic medium (400 mOsm by adding mannitol; shown with a bar on the top). Increased fluorescent signal at 360 nm represents cell shrinking, as indicated by a triangle on the right. The control perfusion medium was 300 mOsm. The number of individual tested cells is shown (N = 2 different mice were used). **(C)** Representative pseudocolor [Ca^2+^]_i_ images (upper panel) and respective Ca^2+^ -independent Fura 2 fluorescence F_360_ (lower panel) in a part of a split-opened CD during perfusion with control 300 mOsm medium (left row), upon application of hypotonic 220 mOsm medium (middle row), and followed by washout with the control medium (right row). Decreased osmolarity was achieved by reducing mannitol concentration from 155 mM (control) to 80 mM (hypotonic). A typical individual region of interest is shown with a dashed circle.

Acute variations in extracellular osmolarity induce inevitable changes in cell volume [41]. Importantly, the rate of initial volume changes is a reflection of cell water permeability serving as an index of aquaporin expression/activity at the plasma membrane [42]. To investigate the significance of TRPC3-dependent [Ca^2+^]_i_ signaling on CD water transport, we next employed simultaneous measurements of [Ca^2+^]_i_ dynamics (the calibrated ratio of Fura-2 emissions upon excitation at 340 nm and 380 nm, F_340_/F_380_) and relative changes of cell volume (by quantifying changes in Fura-2 intensity with excitation at 360 nm (F_360_), the Ca^2+^-independent “isosbestic” point [36]). Firstly, we verified that there are no cross-contaminations between fluorescent-based measurements of [Ca^2+^]_i_ and cell volume. As shown in Fig 2A, removal of extracellular Ca^2+^ (5 mM EGTA) markedly reduced [Ca^2+^]_i_ levels (black), but did not affect the F_360_ signal (grey), suggesting no volume changes under this condition, as expected. Furthermore, application of hypertonic medium (400 mOsm) induced a reversible increase of F_360_ (Fig 2B, grey trace), thus representing a shrinkage of the CD cells. In contrast, hypertonicity did not elicit a measurable [Ca^2+^]_i_ response (Fig 2B, black trace). Indeed, hypotonicity, but not hypertonicity is associated with changes in [Ca^2+^]_i_ in a variety of cell types (reviewed in [41]). Thus, the results in Fig 2 demonstrate reliability of simultaneous measurements of [Ca^2+^]_i_ dynamics and volume in CD cells suggesting no overlap in the respective fluorescent signals.

### Hypotonic [Ca^2+^]_i_ responses and volume regulation are compromised in CDs from TRPC3-/- mice

**Fig 3:**
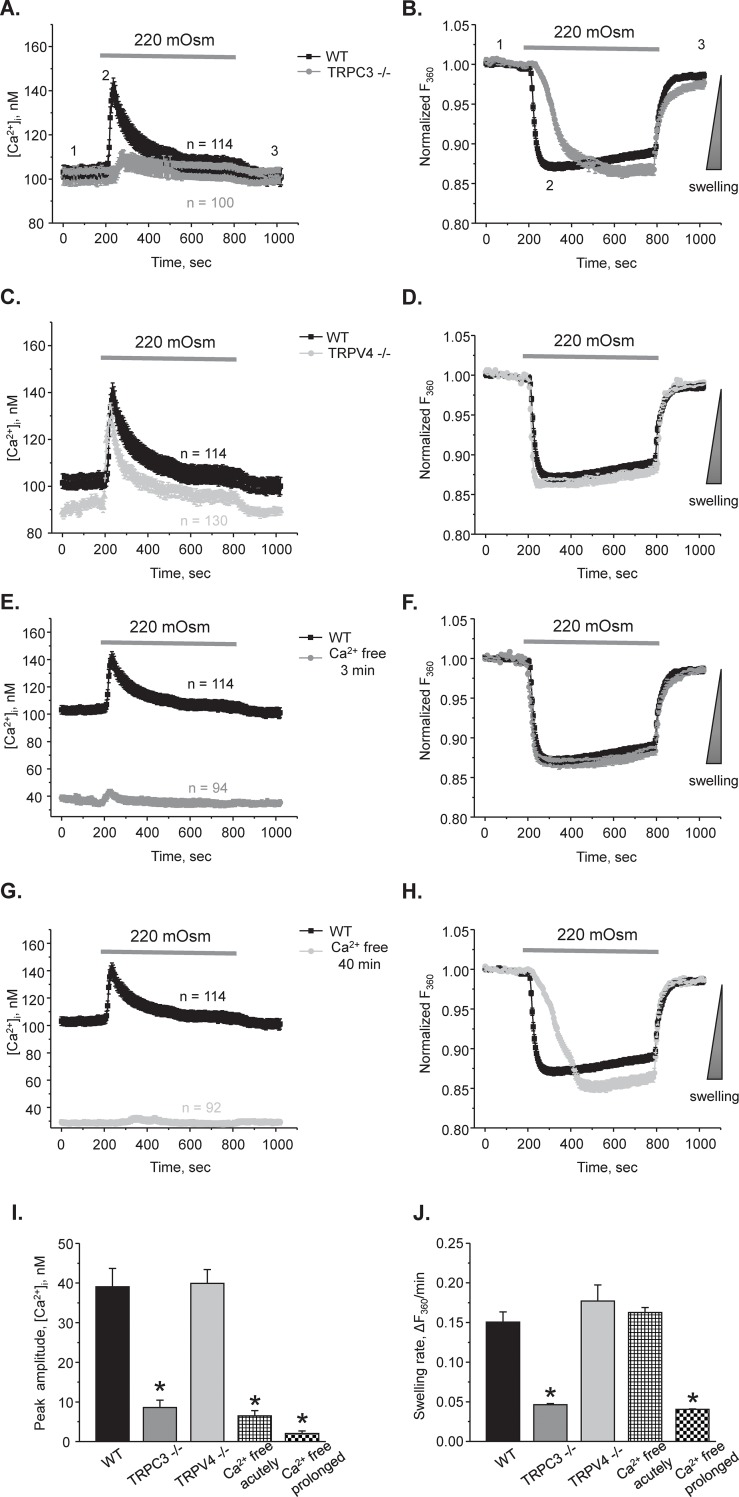
Deletion of TRPC3 but not TRPV4 compromises osmosensitive [Ca^2+^]_i_ signaling and volume regulation in CD cells. The average time courses of changes in [Ca^2+^]_i_ **(A)** and respective relative increases in cell volume **(B)** in response to application of hypotonic 220 mOsm medium in individual cells from split-opened CDs of WT (black) and TRPC3 -/- (grey) mice. Application time is shown with a respective bar on the top. Decreased osmolarity was achieved by reducing mannitol concentration from 155 mM in the control 300 mOsm medium to 80 mM in hypotonic medium. The direction of cell swelling is shown by a triangle on the right in panel B. The average time courses of changes in [Ca^2+^]_i_ **(C)** and respective relative increases in cell volume **(D)** in response to application of hypotonic 220 mOsm medium in individual cells from split-opened CDs of WT (black) and TRPV4 -/- (light grey) mice. The experimental conditions were identical to those described in panels A and B. The average time courses of changes in [Ca^2+^]_i_ **(E)** and respective relative increases in cell volume **(F)** in response to application of hypotonic 220 mOsm medium in individual cells from split-opened CDs of WT mice in the control (black) and after acute 3 min pretreatment with Ca^2+^ free medium (grey). The average time courses of changes in [Ca^2+^]_i_ **(G)** and respective relative increases in cell volume **(H)** in response to application of hypotonic 220 mOsm medium in individual cells from split-opened CDs of WT mice in the control (black) and after prolonged 40 min pretreatment conditions with Ca^2+^ free medium (light grey). For this, freshly isolated split-opened CDs were loaded with Fura 2 in the absence of extracellular Ca^2+^. All other experimental conditions were identical to those described in panels A and B. Summary graphs comparing the average magnitudes of transient peak [Ca^2+^]_i_ responses **(I)** and cell swelling rates **(J)** to application of hypotonic 220 mOsm medium for the tested experimental conditions in the panels A-H. The rate of cell swelling was calculated as a linear slope of the initial changes in Fura 2 fluorescent intensity at 360 nm excitation upon application of osmotic stimuli for each cell individually. *—significant decrease versus WT. N = 3 different mice were used for each experimental group.

Our results (Fig 1C and 1D) indicate that TRPC3-dependent Ca^2+^ entry is a critical determinant of [Ca^2+^]_i_ responses to hypotonicity. Thus, we next investigated the relation between osmosensitive [Ca^2+^]_i_ signaling and cell volume in the CDs from WT and TRPC3 -/- mice. Fig 2C and S1A Fig show high and low magnification representative micrographs from an experiment simultaneously monitoring [Ca^2+^]_i_ levels and relative changes in individual cell volume in the control, during application of a hypotonic medium (220 mOsm) for 10 min, and following washout with 300 mOsm in a CD from a WT mouse. The average time courses of [Ca^2+^]_i_ and relative volume changes are shown in Fig 3A and 3B, respectively. In CDs from WT mice (black traces), the [Ca^2+^]_i_ response to hypotonicity constituted of a transient Ca^2+^ peak followed by a sustained plateau; and the respective cell volume response consisted of a rapid swelling phase followed by a slow volume recovery (i.e. a regulatory volume decrease). In CDs from TRPC3 -/- mice, the [Ca^2+^]_i_ response to hypotonicity was nearly abolished (Fig 3A; see also representative low magnification micrographs in S1B Fig). While the magnitude of cell swelling was comparable between WT and TRPC3 -/- cells, the rate of volume changes was much slower in CDs from TRPC3 -/- mice (Fig 3B). In contrast, CDs from TRPV4 -/- mice exhibited comparable with WT hypotonicity-induced [Ca^2+^]i responses and respective changes in cell volume (Fig 3C and 3D). This is consistent with the data in Fig 1 showing no appreciable role of the TRPV4 channel in mediating osmosensitive [Ca^2+^]_i_ signaling.

Our results in Fig 3A and 3B show that the lack of TRPC3-dependent [Ca^2+^]_i_ influx is associated with slower cell volume changes in response to hypotonicity. It has been established that the rate of swelling in response to hypotonic exposure positively correlates with plasma membrane abundance of aquaporin channels (AQPs), whereas the magnitude of the response is determined by the existing osmotic gradient [42–44]. Thus, we hypothesized that TRPC3 activity is necessary for effective translocation of AQP2 to the apical plasma membrane in CD cells. Acute removal of extracellular Ca^2+^ (5 mM EGTA for 3 min) decreased basal [Ca^2+^]_i_ levels and abolished [Ca^2+^]_i_ response to hypotonicity in CDs from WT mice (Fig 3E). However, it did not change the rate of cell swelling compared to the control conditions (Fig 3F). In contrast, prolonged exposure of the CDs to Ca^2+^ free medium for 40 min precluded osmosensitive [Ca^2+^]_i_ signaling (Fig 3G) and also decreased the rate of cell swelling to the values observed in TRPC3 -/- mice (Fig 3H). The average peak values of osmosensitive [Ca^2+^]_i_ responses and the rates of swelling were summarized in Fig 3I and 3J, respectively. Altogether, our results demonstrate that deletion of TRPC3 but not TRPV4 abolishes [Ca^2+^]_i_ elevations in response to reduced extracellular osmolarity and slows the rate of cell swelling serving as an index of water permeability of the plasma membrane. While both acute and prolonged removal of extracellular Ca^2+^ disrupted [Ca^2+^]_i_ responses to hypotonicity, only prolonged removal of Ca^2+^ recapitulated the phenotype seen in TRPC3 -/- mice thus indicating the inhibitory effect on aquaporin trafficking.

### TRPC3 dysfunction decreases water reabsorption in the CD and diminishes urinary concentrating ability

**Fig 4:**
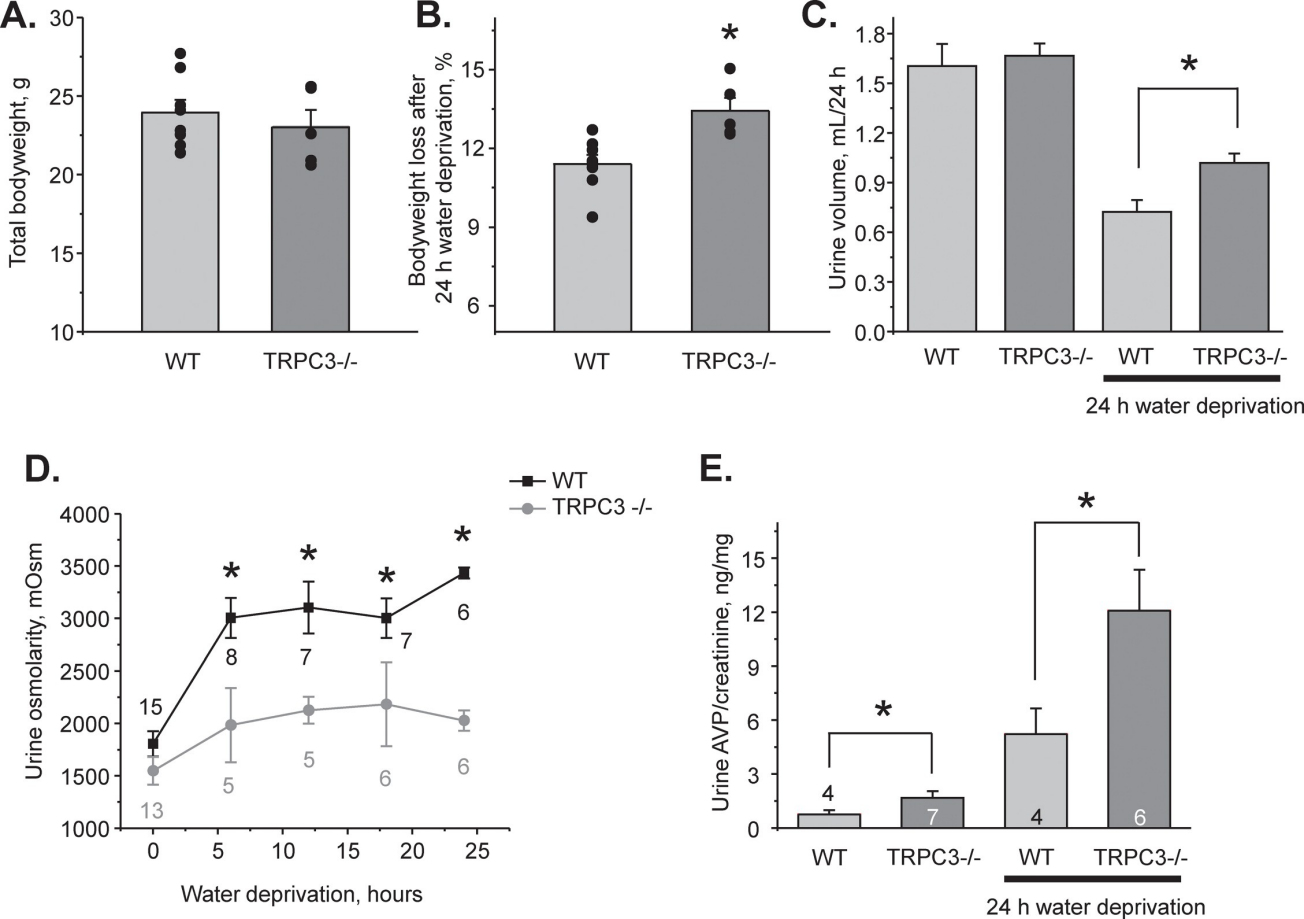
TRPC3 -/- mice develop urinary concentrating defect. **(A)** Summary graph comparing whole body animal weight of WT and TRPC3 -/- mice used for metabolic cage balance studies. **(B)** Summary graph of weight loss induced by 24h water deprivation in WT and TRPC3 -/- mice. **(C)** Summary graph comparing 24h urinary output in WT and TRPC3 -/- mice placed in metabolic cages in the control and after 24h water deprivation. **(D)** Summary graph comparing urinary osmolarities at the baseline and during 6, 12, 18, and 24 hour water deprivation in WT and TRPC3 -/- mice. **(E)** Summary graph of urinary arginine vasopressin (AVP) levels in WT and TRPC3 -/- mice at the baseline and after 24h water deprivation. AVP levels were normalized on the respective urinary creatinine concentrations. Number of tested animals for each experimental time-point is shown. *—significant change versus respective values in WT animals.

Since our results support the view of reduced expression of aquaporins at the plasma membrane, we next examined the significance of TRPC3 for systemic water balance and urinary production using metabolic cage balance studies. There were no differences in total bodyweight of age matched WT and TRPC3 -/- mice at the baseline (Fig 4A). Water deprivation for 24 hours induced a significantly greater weight loss in the knockouts 13.4±0.4% versus 11.4±0.3% in WT (Fig 4B). Urine volume was comparable at the baseline 1.60±0.13 ml/24h (WT) and 1.67±0.07 ml/24h (TRPC3 -/-), but significantly larger in knockouts after 24h water deprivation: 1.01±0.05 ml versus 0.72±0.07 ml in WT (Fig 4C). Consistently, plasma osmolarity was similar 322±3 mOsm (WT) and 326±1 mOsm (TRPC3 -/-) at the baseline, but significantly larger in knockouts after 24h water deprivation: 348±3 mOsm versus 340±3 mOsm in WT (P = 0.04).

At the baseline, urinary osmolarities were modestly reduced in TRPC3 -/- mice compared to WT mice (Fig 4D): 1546 ± 132 mOsm versus 1804 ± 119 mOsm (P = 0.06). This difference was exacerbated during 24h water deprivation suggesting the urinary concentrating defect in TRPC3 -/- mice (Fig 4D). Urinary AVP levels were significantly higher in TRPC3 -/- mice compared to WT mice at the baseline and after 24h water deprivation (Fig 4E) arguing that the concentration defect is associated with impaired renal water conservation and is not due to deficient AVP synthesis/release. Altogether, the studies in Fig 4 demonstrate that TRPC3 deletion disturbs systemic water balance during antidiuretic conditions.

### TRPC3 is necessary for AQP2 translocation/localization to the apical plasma membrane

**Fig 5:**
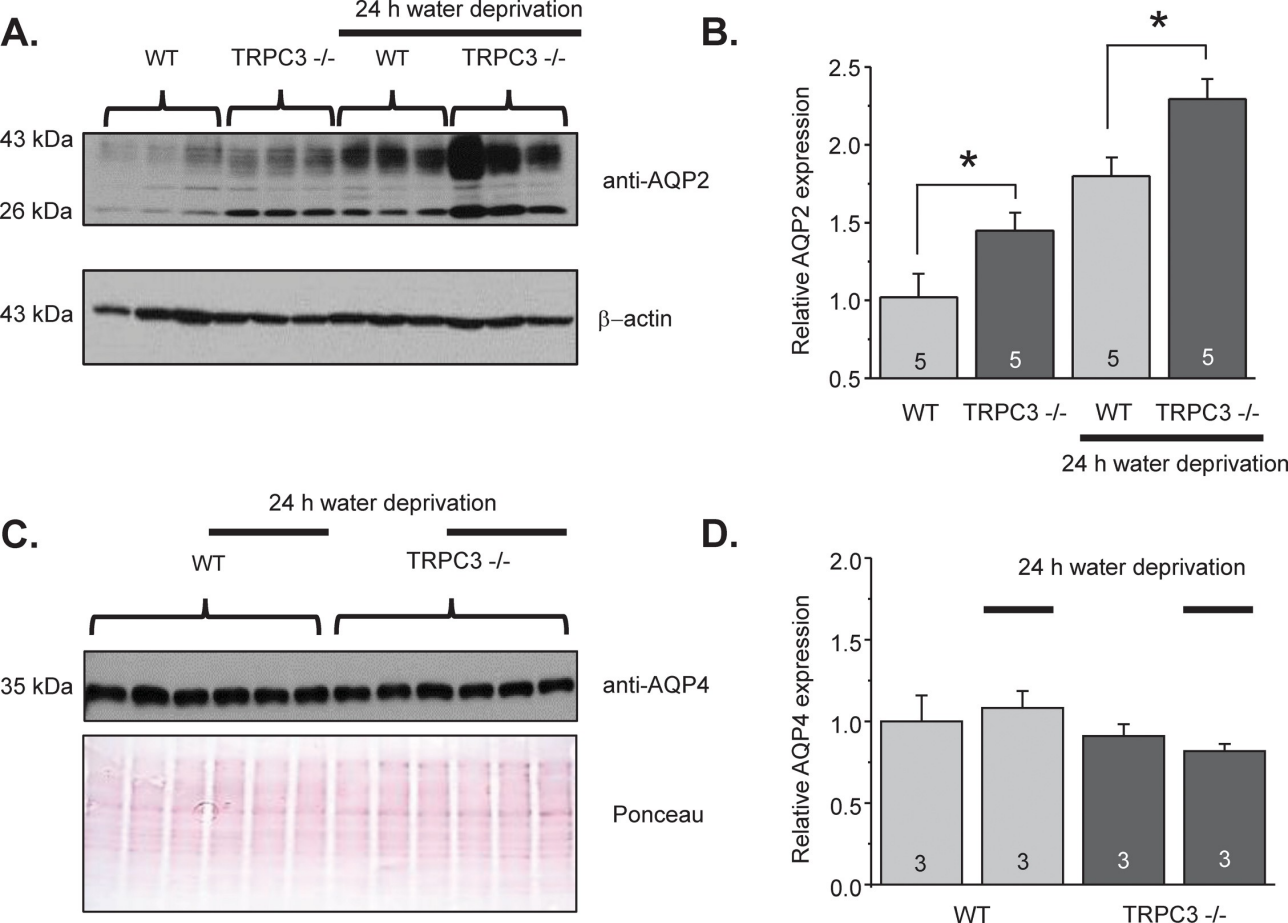
Increased renal abundance of AQP2 but not AQP4 in TRPC3 -/- mice. **(A)** Representative Western blot from whole kidney lysates of WT and TRPC3 -/- mice at the baseline and after 24h water deprivation probed with anti-AQP2 and anti β-actin antibodies. AQP2 appears as a duplet of the upper glycosylated near 37 kDa and lower non-glycosylated near 29 kDa bands. Each kidney was from separate animal. (**B**) Summary graph comparing total renal AQP2 expression (both glycosylated and non-glycosylated forms) in WT and TRPC3 -/- mice from Western blots similar to that shown in (A) Intensities of AQP2-reporting bands were normalized to the intensities of the respective actin bands. *—significant increase versus respective WT values. **(C)** Western blot from whole kidney lysates of WT and TRPC3 -/- mice at the baseline and after 24h water deprivation (indicated by a line) probed with anti-AQP4 antibodies. The Ponceau red staining of the same nitrocellulose membrane demonstrating equal protein loading is shown below. **(D)** Summary graph comparing total renal AQP4 expression for WT and TRPC3 -/- mice under tested conditions. The intensity values were normalized to the total signal of the respective lines in Ponceau red staining.

We next proceeded with examining how TRPC3 deficiency affects AQP2 expression and localization in the CDs. Renal AQP2 levels were moderately but significantly higher TRPC3 -/- mice compared to WT mice, as shown in the representative Western blot in Fig 5A and the summary graph in Fig 5B. Water deprivation for 24h further increased AQP2 expression in WT as well as in TRPC3 -/- mice (Fig 5A and 5B). The pattern of AQP2 expression in WT and TRPC3 -/- mice correlated nicely with the respective AVP levels under these conditions (Fig 4E). At the same time, TRPC3 deletion did not affect AQP4 levels in the control and after water deprivation (Fig 5C and 5D). AQP4 are constitutively expressed at the basolateral membrane in the CD cells and are not regulated by AVP [1]. Thus, our results suggest that TRPC3 regulates apical but not basolateral water permeability in the CD. Furthermore, impaired urinary concentrating ability in the TRPC3 knockouts is likely caused by a defective localization of AQP2 but not changes in AQP2 levels.

**Fig 6:**
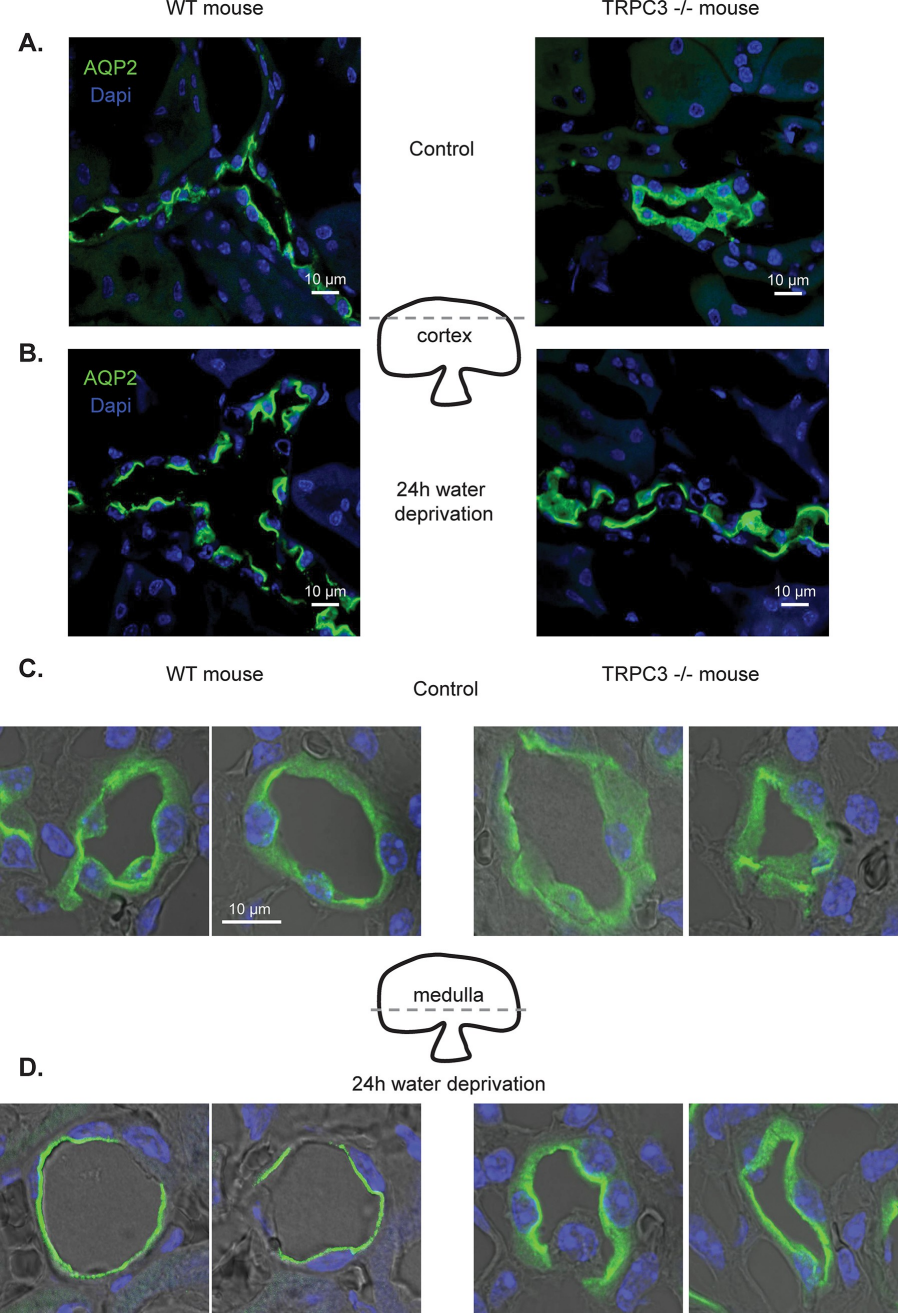
TRPC3 deletion promotes AQP2 retention in the cytosol in the CD cells. Representative high magnification confocal micrographs of cortical kidney sections probed with anti-AQP2 (pseudocolor green) and nuclear Dapi (pseudocolor blue) from WT and TRPC3 -/- in the control **(A)** and after 24h water deprivation **(B)**. Representative high magnification confocal micrographs of medullary kidney sections probed with anti-AQP2 (pseudocolor green) and nuclear Dapi (pseudocolor blue) from WT and TRPC3 -/- in the control **(C)** and after 24h water deprivation **(D)**. The approximate position of tested planes for cortical and medullary sections is outlined on the middle. The micrographs are representative of captured 10 different CDs from 5 kidneys.

We next visualized subcellular distribution of the AQP2-reporting fluorescent signals in kidney sections from WT and TRPC3 -/- mice in the control and after 24h water deprivation to stimulate AVP signaling. As shown on the high magnification representative micrographs of individual cortical CDs, AQP2 was largely distributed in the cytosol in TRPC3 -/- mice compared to AQP2 localization to the apical region in WT animals (Fig 6A). The images were typical of ~10 examined CDs from 5 different kidneys for each condition (see also S2 Fig for overall view of the renal sections). Furthermore, AQP2-reporting signal was further accumulated in the apical regions of cortical CD cells from WT mice after 24h water deprivation (Fig 6B). We also observed moderate AQP2 translocation towards the apical plasma membrane in CD cells from water deprived TRPC3 -/- mice, but a substantial portion of AQP2 remained in the cytosol (Fig 6B). Similarly, the compromised AQP2 distribution and apical translocation in response to water deprivation was observed in medullary CDs from TRPC3 -/- mice (Fig 6C and 6D). Altogether, these results suggest that TRPC3 dysfunction interferes with AVP-induced AQP2 trafficking to the apical plasma membrane in both cultured and native CD cells.

**Fig 7:**
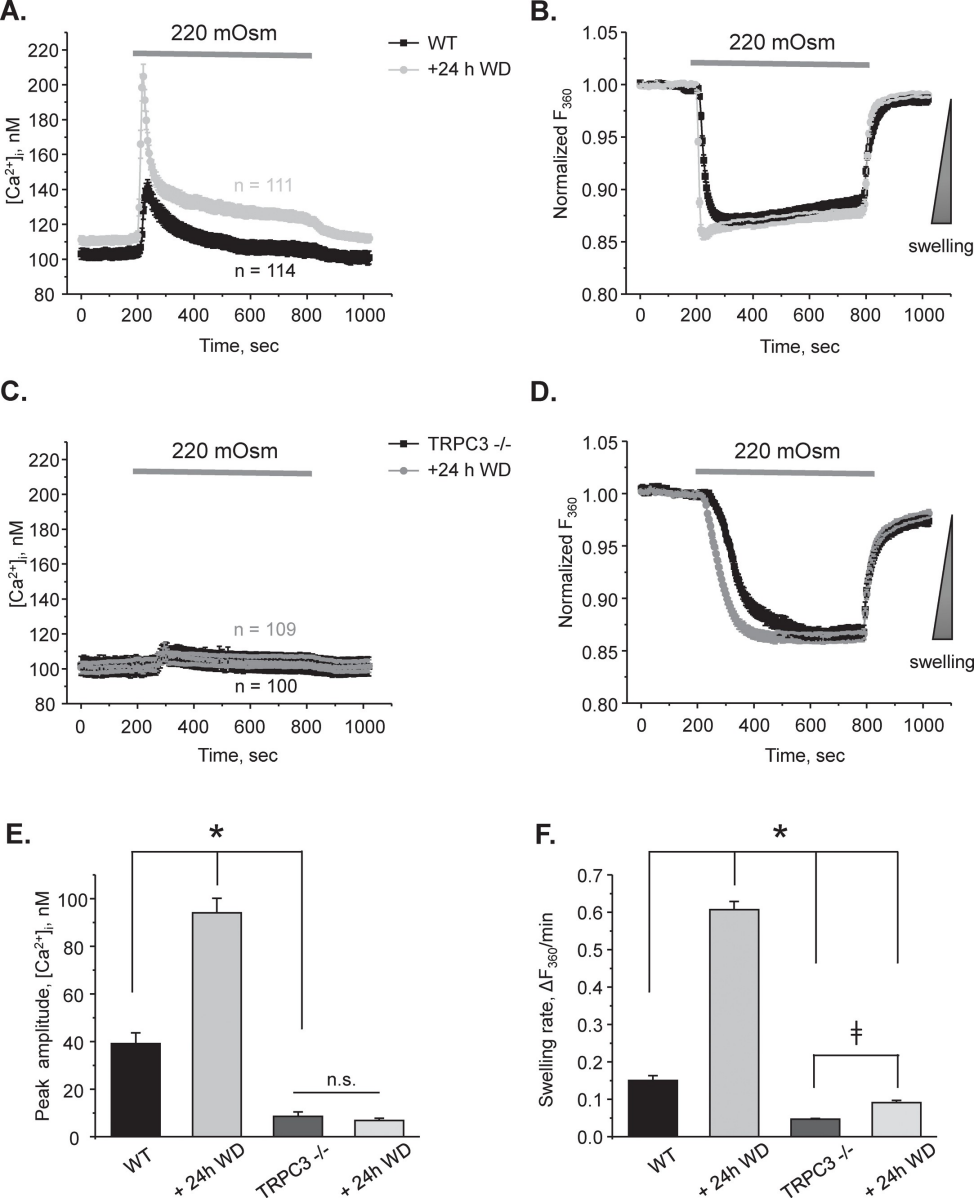
Osmosensitive [Ca^2+^]_i_ signaling and cell volume regulation are compromised in CDs from TRPC3 -/- mice after 24h water deprivation. The average time courses of changes in [Ca^2+^]_i_ **(A)** and respective relative increases in cell volume **(B)** in response to application of hypotonic 220 mOsm medium in individual cells from split-opened CDs of WT mice in the control (black) and after water deprivation (WD) for 24 hours (grey). Application time is shown with a respective bar on the top. Decreased osmolarity was achieved by reducing mannitol concentration from 155 mM in the control 300 mOsm medium to 80 mM in hypotonic medium. The direction of cell swelling is shown by a triangle on the right in panel B. The average time courses of changes in [Ca^2+^]_i_ **(C)** and respective relative increases in cell volume **(D)** in response to application of hypotonic 220 mOsm medium in individual cells from split-opened CDs of TRPC3 -/- mice in the control (black) and after water deprivation (WD) for 24 hours (grey). Application time is shown with a respective bar on the top. All other experimental conditions were identical to those described in panels A and B. Summary graphs comparing the average magnitudes of transient peak [Ca^2+^]_i_ responses **(E)** and cell swelling rates **(F)** to application of hypotonic 220 mOsm medium for the tested experimental conditions in the panels A-D. The rate of cell swelling was calculated as a linear slope of the initial changes in Fura 2 fluorescent intensity at 360 nm excitation upon application of osmotic stimuli for each cell individually. N.S.—not significant. * or ǂ- significant changes between groups as is shown with brackets. N = 3 different mice were used for each experimental group.

Finally, we isolated CDs from WT and TRPC3 -/- animals after 24h water deprivation to monitor osmosensitive volume changes as an index of functional AQP2 at the apical plasma membrane. Application of hypotonic medium drastically augmented [Ca^2+^]_i_ responses and further accelerated rate of cell swelling when compared to the control conditions in WT mice (Fig 7A and 7B). In contrast, hypotonicity elicited only a miniscule [Ca^2+^]_i_ response in CD cells from TRPC3 -/- animals, which was indistinguishable from that observed in the control (Fig 7C). This suggests that there is no additional pathway to induce osmosensitive [Ca^2+^]_i_ response in CD cells during water deprivation. Fig 7E shows the summary of the peak amplitude of the osmosensitive [Ca^2+^]_i_ response in WT and TRPC3 -/- mice under tested conditions. Of note, we found a moderately accelerated cell swelling in CD from TRPC3 -/- water deprived mice (Fig 7D). However, the rate of cell swelling was increased by approximately 2 fold in TRPC3 -/- as opposed to 4 fold potentiation in WT after water deprivation (Fig 7F). Altogether, our results directly demonstrate a defective AQP2 trafficking and reduced water permeability in the CDs from TRPC3 -/- mice.

## Discussion

**Fig 8:**
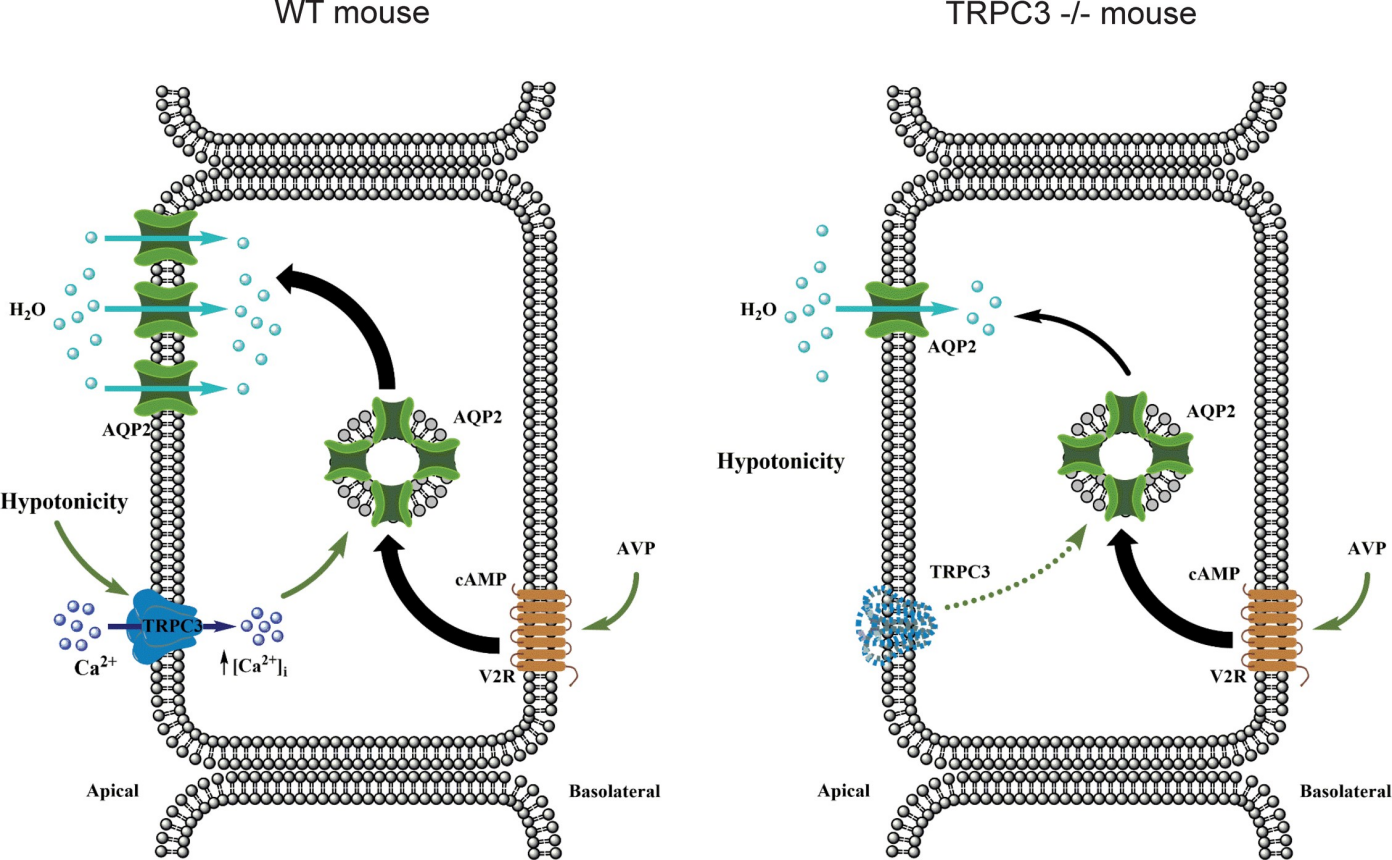
Principal scheme of osmosensitive TRPC3-dependent Ca^2+^ influx into regulation of AQP2 mediated water transport in the CD. cAMP–cyclic adenosine monophosphate, V2R –type 2 vasopressin receptors, AVP–arginine vasopressin. TRPC3 deletion does not interfere with AVP-V2R induced AQP2 synthesis. In contrast, the lack of osmosensitive Ca^2+^ flux via TRPC3 diminishes AQP2 trafficking to the apical plasma membrane in mutant mice.

Based on the results of this study, we propose a model where TRPC3 serves as a convergent point between osmosensitive [Ca^2+^]_i_ signaling and AQP2-water transport in the CD to regulate renal water handling (Fig 8). Activation of TRPC3 by reduced luminal osmolarity leads to elevation of [Ca^2+^]_i_, which is an important signal for redistribution of AQP2-containing vesicles to the apical plasma membrane in the presence of AVP to increase water permeability in the CD. We further propose that TRPC3 participates in a feedback mechanism controlling AQP2 abundance at the apical plasma membrane during antidiuresis. Reduced tonicity of the luminal fluid stimulates TRPC3-dependent Ca^2+^ influx favoring trafficking and incorporation of additional AQP2 to the apical plasma membrane to augment water reabsorption. In contrast, the lack of osmotic gradient and subsequently no TRPC3 activation would indicate that the apical plasma membrane has sufficient levels of AQP2. This precludes further incorporation of AQP2, since it would not stimulate water reabsorption. Disruption of this mechanism in TRPC3 -/- mice results in inappropriate AQP2 retention in the cytosol even in the presence of an augmented AVP signal leading to defective antidiuretic response and decreased urinary concentrating ability.

This manuscript provides an important advance in our understanding of the molecular mechanisms of mechanosensitivity in the distal renal nephron. Using native CD cells from genetically manipulated animals, we demonstrate that distinct types of mechanical stress, namely flow-mediated shear stress and hypotonicity-induced cell swelling, elicit [Ca^2+^]_i_ elevations in the CD cells via distinct pathways (Figs 1 and 3). Whereas Ca^2+^ signaling due to elevated tubular flow is governed by the activity of the Ca^2+^-permeable TRPV4 channel, decreases in extracellular osmolarity (hypotonicity) stimulate the activity of another Ca^2+^-permeable channel, TRPC3. This argues that the CD cells are able to discriminate between specific types of mechanical stress on the plasma membrane by triggering a proper Ca^2+^-dependent response. Consistently, it was shown that fluid shear stress and circumferential stretch differentially affect ERK and p38 activation and PGE2 release in cortical CD cells [45]. It also appears that these pathways operate independently. Thus, disruption of sensitivity to one type of mechanical stress, such as high flow, does not affect osmosensitive [Ca^2+^]_i_ elevations. Indeed, while TRPV4 -/- mice demonstrate impaired sensitivity to renal tubular flow and have defective flow-induced K^+^ secretion in the CD [18, 38], they have intact responses to hypotonicity (Figs 1 and 3) and do not exhibit alterations in renal water transport and urinary production [20, 21]. One may ask how specificity of cellular responses could be achieved if activation of either TRPC3 or TRPV4 leads to global elevations of [Ca^2+^]_i_ in CD cells. We can speculate that this is dictated by drastically different physiological conditions leading to activation of a respective channel. Thus, TRPV4-dependent Ca^2+^ influx would not increase apical water permeability, since AVP levels are low under conditions associated with increased tubular flow, such as high K^+^ diet. Similarly, TRPC3-dependent Ca^2+^ influx would not notably stimulate K^+^ secretion during antidiuresis, since tubular flow is not elevated as necessary for kaliuresis. It has to be noted though, that inhibition of Ca^2+^ release from the endoplasmic reticulum with thapsigargin also has a mild but significant effect on the magnitude of osmosensitive [Ca^2+^]_i_ responses (Fig 1C). It appears that the initial TRPC3-dependent Ca^2+^ influx could be further amplified by the secondary the Ca^2+^ release from the intracellular stores via the calcium- induced-calcium-release (CICR) mechanism, as was similarly reported for TRP channels [46]. In agreement, the removal of extracellular Ca^2+^ abolished osmosensitive [Ca^2+^]_i_ responses in CD cells (Fig 3).

From the methodological standpoint, we demonstrated the possibility to concomitantly monitor changes in [Ca^2+^]_i_ and volume in CD cells within a split-opened area with no cross-contamination between the measured signals (Fig 2). Our experimental setup had enough temporal resolution to detect alterations in volume dynamics in response to genetic and pharmacological interventions and systemic water deprivation (Figs 3 and 7). Importantly, this allowed us to establish a functional link between osmosensitive [Ca^2+^]_i_ signaling and water permeability of the native CD cells, since the rate of initial cell volume changes depends on the abundance of aquaporins in the plasma membrane [41, 42]. Previous studies pointed to technical limitations of fluorescent-based volume measurements particularly in monolayers. This is attributed primarily to an uneven distribution of the fluorescent signals in the center and periphery of a cell, as well as a non-uniform pattern of cell swelling due to spatial restrictions by the neighboring cells [42, 47]. The split-opened CD preparations do not seem to suffer from these problems. Specifically, we did not detect noticeable compartmentalization of Fura 2 signals in the cells (Fig 2). Application of hypotonicity elicited a uniform decrease in Fura 2 fluorescence with 360 nm excitation (Ca^2+^ insensitive point) similarly in the center and periphery (Fig 2C, middle panel). Moreover, the magnitude of measured changes did not depend on a selection of smaller regions of interests within the cell (not shown). At the same time, there are apparent differences in cellular volume changes induced by hypotonicity in the split-open CDs of this study and perfused rabbit CDs [48]. Using quantitative light microscopy, it was shown that CD cells exhibited almost no volume changes in response variations in luminal osmolarity. This was attributed to a substantially greater water permeability of the basolateral membrane [48] likely due to constitutive expression of AQP3/AQP4 [49]. Instead, blockade of the basolateral water conductance with silicone oil induced prominent swelling of the perfused CDs [48], as is observed in our study (Fig 2). This suggests that split-open preparation is effective in limiting access to the basolateral membrane (at least acutely) and the detected changes in cell swelling dynamics largely reflect alterations in the apical water permeability, while contribution of the basolateral membrane cannot be discarded. Indeed, we detected a significant acceleration of the cell swelling in response to 24h water deprivation (Fig 7). This cannot be attributed to the putative changes in basolateral membrane water permeability, since neither AQP3 nor AQP4 are regulated by AVP and we did not detect any changes in AQP4 levels in WT and TRPC3 -/- mice at the baseline and followed by 24h water deprivation (Fig 5C and 5D).

The major finding of this study is that activation of TRPC3 by luminal hypotonicity plays an important role in AQP2 trafficking to the apical plasma membrane. Augmented TRPC3-mediated [Ca^2+^]_i_ responses were associated with accelerated CD cell swelling rates in response to hypotonicity (Fig 7A and 7B). On the other side, TRPC3 deletion (Fig 3A and 3B) or ablation of TRPC3-dependent Ca^2+^ influx in WT mice (Fig 3G and 3H) result in diminished water permeability of the CD cells. Importantly, TRPC3 dysfunction disturbs systemic water balance and diminishes urinary concentrating ability even in the presence of a stronger antidiuretic AVP signal (Fig 4). This correlates nicely with the previously published observation that systemic administration of AVP promotes apical TRPC3 translocation via activation of a cAMP-PKA-dependent pathway [26, 27]. Consistently, we observed augmented TRPC3-mediated [Ca^2+^]_i_ responses in CD cells from WT mice after water deprivation (Fig 7A and 7B). It should be noted that, while our results strongly suggest the involvement of TRPC3 in the CD, extrarenal factors could also contribute to the distorted regulation of water balance upon global TRPC3 deletion. Interestingly, AVP promotes TRPC3 trafficking to the plasma membrane independently of AQP2 [26]. This might indicate an additional role for the channel, independent of controlling water transport. While commonly neglected, the CD is capable to reabsorb Ca^2+^ [50, 51]. Stimulation of TRPC3 promotes transcellular Ca^2+^ flux in cultured IMCD-3 cells [52]. Our results (S3 Fig) also found an increased urinary calcium levels in TRPC3 -/- mice after water deprivation. Thus, it is possible that an AVP-induced TRPC3 translocation would augment CD Ca^2+^ reabsorption to decrease a risk of nephrocalcinosis and urolithiasis by preventing Ca^2+^ precipitation in the luminal fluid during robust water reabsorption in the CD. Consistently, the Williams-Beuren syndrome (WBS), a neurodevelopmental disorder commonly associated with hypercalcemia of unknown origin, has been recently linked to upregulation of TRPC3 expression due to deletion of the gene encoding the transcription factor TFII-I [53]. TFII-I suppresses cell-surface accumulation of TRPC3. Channel expression was high in the CD cells of human kidney biopsies and it was markedly upregulated in intestinal cells of WBS patients, potentially implicating TRPC3 in regulation of systemic Ca^2+^ homeostasis [53]. Future studies are needed to investigate the contribution of TRPC3 in renal Ca^2+^ handling.

In summary, we revealed a previously unrecognized role of the TRPC3 channel in the CD in regulation of AVP-induced water reabsorption and examined pathophysiological consequences of its disruption on AQP2 trafficking at the cellular level and on urinary production/concentration at the whole body level. It is plausible to speculate that TRPC3 inhibition might be instrumental in blocking excessive renal water retention during the clinically relevant states associated high AVP levels, such as congestive heart failure or syndrome of inappropriate AVP secretion [1]. On the contrary, TRPC3 stimulation might be of use to facilitate translocation to the plasma membrane of NDI-causing AQP2 mutants associated with defective trafficking [54].

## Supporting information

S1 Fig Simultaneous monitoring of [Ca^2+^]_i_ and volume in CD from WT and TRPC3 -/- mice. Representative pseudocolor [Ca^2+^]_i_ images (upper panel) and respective Ca^2+^ -independent Fura 2 fluorescence F_360_ (lower panel) in a split-opened CD from WT **(A)** and TRPC3 -/- **(B)** mice during perfusion with control 300 mOsm medium (left row), upon application of hypotonic 220 mOsm medium (middle row), and followed washout with the control medium (right row).
(TIFF)

S2 Fig Immunofluorescent visualization of AQP2 levels in WT and TRPC3 -/- mice. Low magnification images of transverse cut kidney sections from WT **(A)** and TRPC3 **(B)** mice in the control (top) and after 24 hour water deprivation (bottom) used to visualize subcellular AQP2 localization, as shown in Fig 6. AQP2 and nuclear Dapi staining is shown with pseudocolor green and blue, respectively.
(TIFF)

S3 Fig TRPC3 deletion precludes reductions in urinary Ca^2+^ levels in response to water deprivation. Summary graph of spot urinary Ca^2+^ levels in the baseline and after 12h water deprivation in WT and TRPC3 -/- mice. Urinary Ca^2+^ levels were normalized on the respective osmolarities. *—significant decrease versus WT basal.
(TIFF)

S4 Fig Original uncropped Western blots shown in Fig 5A and 5C. (TIFF)
