# Supplementary figures and images for: TRPC3 determines osmosensitive [Ca2+]i signaling in the collecting duct and contributes to urinary concentration
Source: PLoS One. 2019 Dec 18;14(12):e0226381. doi: 10.1371/journal.pone.0226381 (PMC6919627; doi:10.1371/journal.pone.0226381)

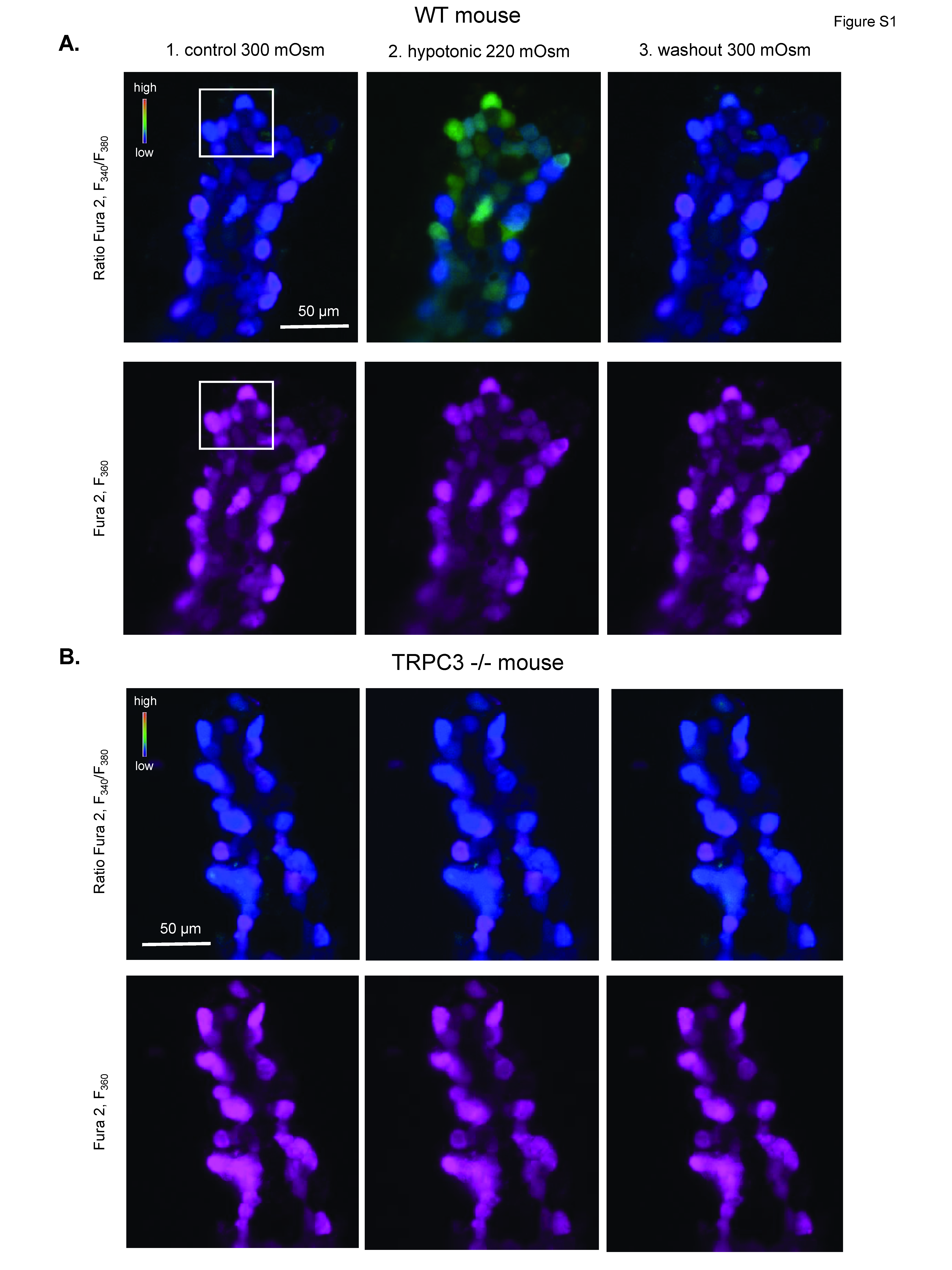

Supplement: S1 Fig — Representative pseudocolor [Ca2+]i images (upper panel) and respective Ca2+ -independent Fura 2 fluorescence F360 (lower panel) in a split-opened CD from WT (A) and TRPC3 -/- (B) mice during perfusion with control 300 mOsm medium (left row), upon application of hypotonic 220 mOsm medium (middle row), and followed washout with the control medium (right row). (TIFF) [file pone.0226381.s001.tiff]

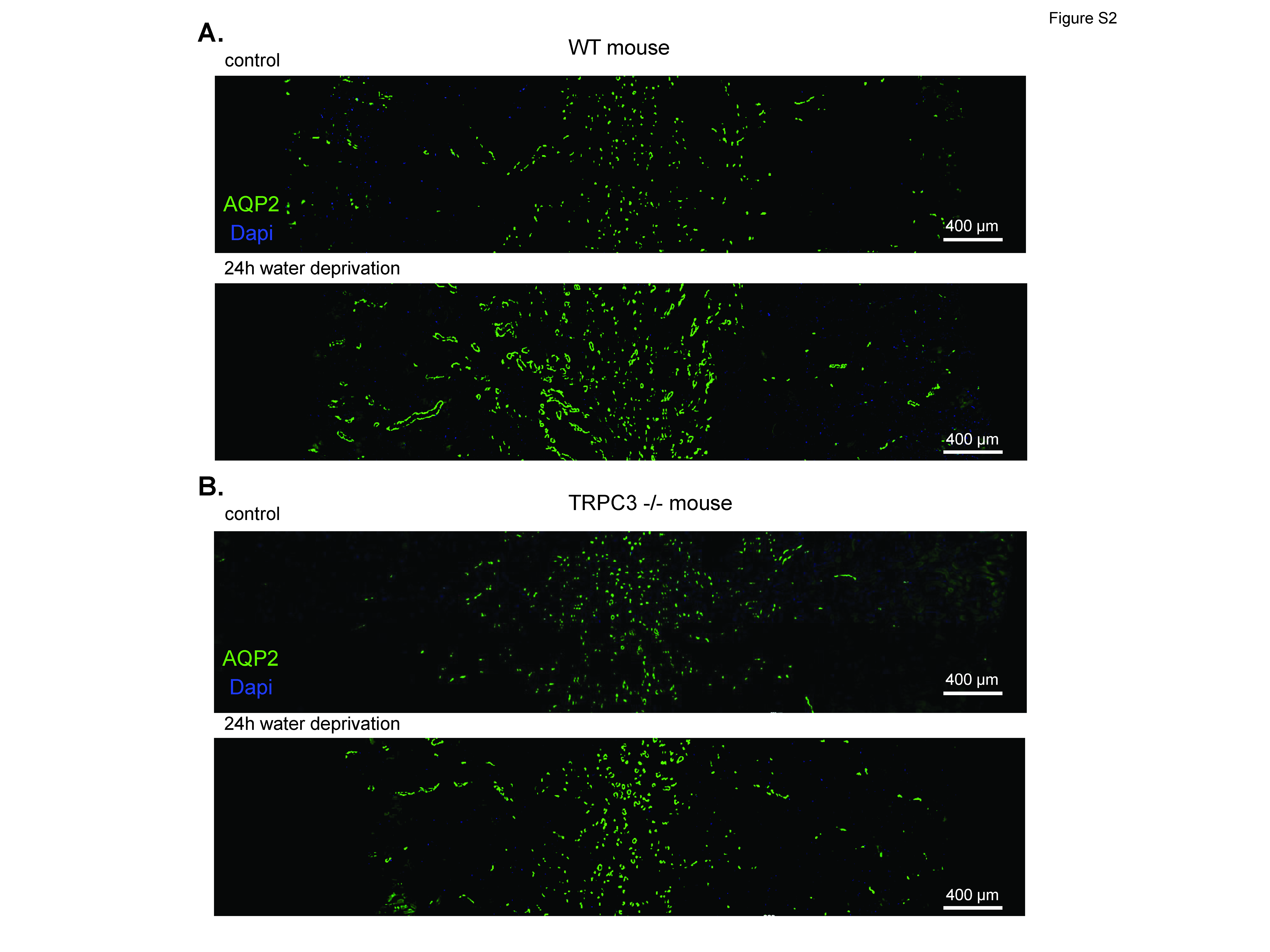

Supplement: S2 Fig — Low magnification images of transverse cut kidney sections from WT (A) and TRPC3 (B) mice in the control (top) and after 24 hour water deprivation (bottom) used to visualize subcellular AQP2 localization, as shown in Fig 6. AQP2 and nuclear Dapi staining is shown with pseudocolor green and blue, respectively. (TIFF) [file pone.0226381.s002.tiff]

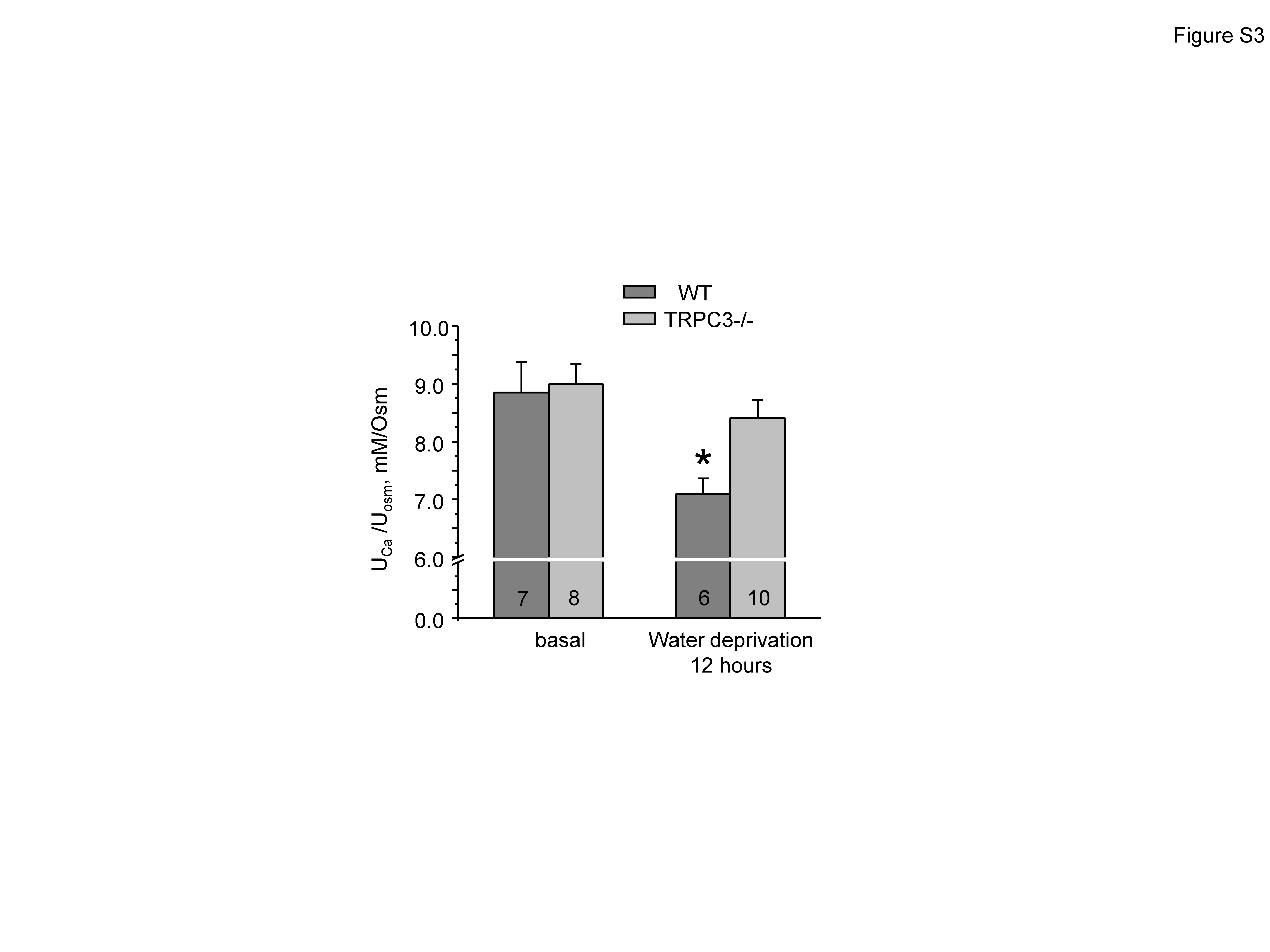

Supplement: S3 Fig — Summary graph of spot urinary Ca2+ levels in the baseline and after 12h water deprivation in WT and TRPC3 -/- mice. Urinary Ca2+ levels were normalized on the respective osmolarities. *—significant decrease versus WT basal. (TIFF) [file pone.0226381.s003.tiff]

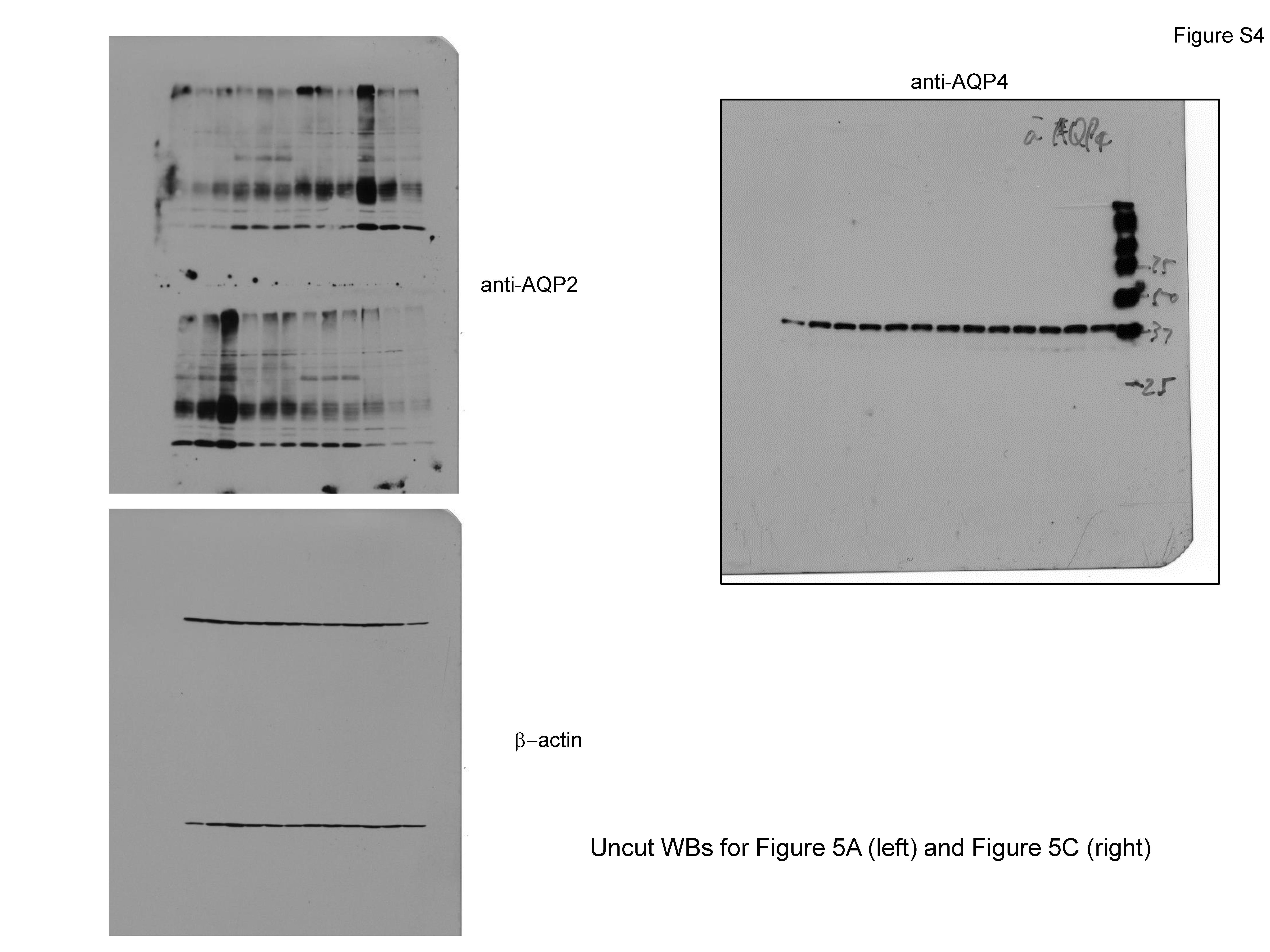

Supplement: S4 Fig — (TIFF) [file pone.0226381.s004.tiff]
